# Single amino acids in sucrose rewards modulate feeding and associative learning in the honeybee

**DOI:** 10.1016/j.jinsphys.2014.05.004

**Published:** 2014-10

**Authors:** Nicola K. Simcock, Helen E. Gray, Geraldine A. Wright

**Affiliations:** Institute of Neuroscience, Newcastle University, Newcastle upon Tyne NE1 7RU, United Kingdom

**Keywords:** Honeybee, Amino acids, Olfactory learning, Nutrient balancing, Gustation

## Abstract

Obtaining the correct balance of nutrients requires that the brain integrates information about the body’s nutritional state with sensory information from food to guide feeding behaviour. Learning is a mechanism that allows animals to identify cues associated with nutrients so that they can be located quickly when required. Feedback about nutritional state is essential for nutrient balancing and could influence learning. How specific this feedback is to individual nutrients has not often been examined. Here, we tested how the honeybee’s nutritional state influenced the likelihood it would feed on and learn sucrose solutions containing single amino acids. Nutritional state was manipulated by pre-feeding bees with either 1 M sucrose or 1 M sucrose containing 100 mM of isoleucine, proline, phenylalanine, or methionine 24 h prior to olfactory conditioning of the proboscis extension response. We found that bees pre-fed sucrose solution consumed less of solutions containing amino acids and were also less likely to learn to associate amino acid solutions with odours. Unexpectedly, bees pre-fed solutions containing an amino acid were also less likely to learn to associate odours with sucrose the next day. Furthermore, they consumed more of and were more likely to learn when rewarded with an amino acid solution if they were pre-fed isoleucine and proline. Our data indicate that single amino acids at relatively high concentrations inhibit feeding on sucrose solutions containing them, and they can act as appetitive reinforcers during learning. Our data also suggest that select amino acids interact with mechanisms that signal nutritional sufficiency to reduce hunger. Based on these experiments, we predict that nutrient balancing for essential amino acids during learning requires integration of information about several amino acids experienced simultaneously.

## Highlights

• The mouthparts are more sensitive to amino acids in sucrose solution than the antennae.
• Pre-feeding with sucrose solutions containing single amino acids reduces learning performance and feeding.
• Iso-leucine was rewarding in sucrose solution whereas methionine was aversive.
• Nutritional state modulated how bees responded to solutions containing proline or phenylalanine.

## Graphical abstract

**Figure:**
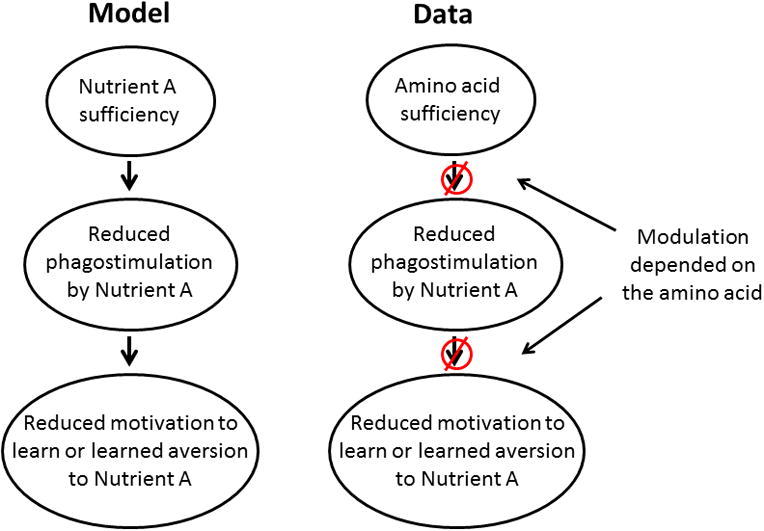

## 1 Introduction

Learning about food is a mechanism that provides animals with flexibility in food choices that improves chances of survival. The taste of food is an important cue used during learning to identify palatable and nutritious foods. Insects detect nutrients like sugars and amino acids (AAs) using sensory neurons housed in sensilla on the mouthparts, antennae, and feet (Scott et al., 2001, Amrein and Thorne, 2005). During appetitive learning, information from taste sensilla is integrated in the brain with visual, olfactory, and tactile cues to form a learned association (Hammer, 1993). This information can be further modulated by cues that arise after food has been consumed. For example, when nutrients have been consumed in association with taste cues, animals form lasting memories of food (Sclafani and Ackroff, 1994, Burke and Waddell, 2011). The taste of food is also an important cue associated with the post-ingestive consequences of eating toxins or unsuitable foods (Garcia et al., 1955, Behmer et al., 1999, Zhang et al., 2005, Wright et al., 2010, Simoes et al., 2012).

Nutrient balancing is a complex process in which animals integrate taste cues with post-ingestive information about food quality to obtain optimal nutrition (Simpson and Raubenheimer, 1997). Most studies of associative learning use carbohydrates to reward animals, but animals can also learn to associate chemical cues like odours and tastes with the presence of protein or AAs in food (Simpson and White, 1990, Raubenheimer and Tucker, 1997). During nutrient balancing, the body detects deficiencies in nutrition and modulates food intake to identify and consume foods that satisfy this deficiency. When AAs or proteins are deficient in diet, insects such as locusts and Drosophila have greater taste sensitivity towards solutions containing AAs (Abisgold and Simpson, 1987, Simpson et al., 1991, Toshima and Tanimura, 2012). Deficiencies also influence how quickly animals learn cues associated with appropriate nutrients. Locusts are more likely to learn to associate odours and tastes with protein in food if they are protein-deficient (Raubenheimer and Tucker, 1997).

It remains unclear whether nutritional oversufficiency can also influence learning. Just as appetitive learning is enhanced by deficiencies, it is possible that aversion learning could be driven by too much of specific nutrients as over consumption of nutrients can be metabolically costly to animals (Zanotto et al., 1994, Zanotto et al., 1997). Too much protein or essential AAs in diet, for example, has high costs for social insect workers, as it decreases lifespan (Pirk et al., 2010, Dussutour and Simpson, 2012, Paoli et al., 2014). If animals are forced to consume foods that are nutritionally deficient in specific essential AAs but sufficient in others, they learn to avoid these foods (Simpson et al., 1991, Koehnle et al., 2003, Toshima and Tanimura, 2012). However, few studies have identified whether nutritional oversufficiency produces learned aversions towards specific nutrients such as individual AAs when they are overabundant.

Honeybees learn to associate visual and olfactory cues with food during foraging for nectar and pollen on flowers, and for this reason, have become an important model system for the study of learning and memory (Menzel, 1983, Hammer and Menzel, 1995). Foraging worker honeybees collect floral nectar but also use it for their own nutritional needs. Floral nectar contains the sugars, sucrose, glucose and fructose, but also contains essential and non-essential AAs (Baker and Baker, 1973, Nicolson et al., 2007). High concentrations of AAs are toxic to honeybee foragers but low concentrations are nutritionally important (Paoli et al., 2014). Few studies of appetitive learning in bees have used AAs in rewards during associative learning, and most of these have concluded that the honeybee’s responses towards AAs are often indistinguishable from a sugar solution (Inouye and Waller, 1984, Kim and Smith, 2000). One study showed that the forager honeybee’s intake of proline, a non-essential AA used by bees as fuel for flight commonly found in nectar (Micheu et al., 2000, Suarez et al., 2005) is modulated by its concentration in solution (Carter et al., 2006), such that bees preferred concentrations around 6 mM and consumed significantly less of sucrose solution containing 100 mM proline. It remains unclear whether the bees in this study were learning to avoid the 100 mM solution or whether they simply avoided drinking it because it tasted repellent.

Here, we tested how nutritional state affected the taste of specific AAs (isoleucine, proline, phenylalanine, and methionine) and whether or not bees learned to avoid relatively high (100 mM) concentrations of these AAs in sucrose solutions. We first tested how feeding with sucrose solution containing a specific AA influenced whether or not bees would consume the solution the next day. We also tested how being fed with sucrose solutions containing individual AAs influenced the honeybee’s rate of learning when these solutions were used as the reward during an olfactory appetitive conditioning task.

## 2 Methods

### 2.1 Subjects

Returning forager honeybees (*Apis mellifera Buckfast*) were collected from a population developed at the National Bee Unit (York, UK). They were captured at a hive entrance between the months of March–July 2011 at Newcastle University and restrained as described in Wright and Smith (2004). They were anesthetized on ice for ∼3 min in glass vials, and then placed in a restraining harness. Bees were restrained within 30 min of catching them at the colony entrance. Restrained bees were used in the feeding assay and in the learning assay. In these assays, bees were commonly fed to satiety (i.e. until they would no longer consume solution or lift the proboscis to feed when stimulated on the antennae). Feeding was accomplished using a 0.2 ml Gilmont micrometer syringe (Gilmont Instruments). To feed the bees, each was tapped on the antennae briefly with 1 M sucrose solution to elicit proboscis extension and then fed 0.4 μl droplets on the proboscis until each bee would no longer consume the solution (i.e. satiety).

### 2.2 Solutions

Solutions of 1 M sucrose and 1 M sucrose with 100 mM of a single amino acid (isoleucine, methionine, phenylalanine or proline, powdered forms, Sigma–Aldrich) were made using distilled water.

### 2.3 Influence of AA solutions on feeding

Bees were restrained as above. A one hour after they were restrained, they were fed to satiety with one of the following solutions: 1 M sucrose or a 1 M sucrose solution containing 100 mM of the following AAs: isoleucine, proline, phenylalanine, or methionine. The volume required to produce satiety was measured; bees generally achieved this within 1–2 min of the start of feeding. After 24 h, each bee was fed to satiety again using the same solution they had been fed the day before. The volume required to produce satiety was measured.

We also tested whether the AAs elicited PER on their own when applied to the mouthparts. Bees were restrained as above and fed 1 M sucrose to satiety. After 24 h, each bee was tapped on the antenna with 1 M sucrose and then fed to satiety using one of the compounds in solution: sucrose, isoleucine, phenylalanine, proline, or methionine. The following concentrations were tested: 100 μM, 1 mM, 10 mM, 100 mM. Separate groups of bees (*N* = 30 each) were tested with each concentration.

### 2.4 Olfactory conditioning protocol

Subjects for the olfactory PER conditioning assay were restrained as above and fed to satiety with either 1 M sucrose or with 1 M sucrose containing 100 mM of isoleucine, methionine, proline, or phenylalanine and left for ∼18–24 h. Immediately prior to conditioning, bees were tested for responsiveness to feed by touching both antennae with 1 M sucrose to elicit PER; those that failed to respond were not used in the conditioning assay. Subsequently, each subject underwent a 12 trial olfactory conditioning paradigm of the PER (Bitterman et al., 1983, Wright et al., 2010) with a 5 min inter-trial interval (ITI). The conditioned stimulus (CS) was a 4 s odour pulse controlled by a programmable logic controller; the odour was delivered via a 5 mm × 75 mm glass tube containing filter paper covered with a 3 μl aliquot of pure odour solution (1-hexanol, Sigma–Aldrich). The unconditioned stimulus (US) was presented approximately 3 s after the start of the CS. The US was a 0.4 μl droplet of experimental solution (1 M sucrose or 100 mM AA in 1 M sucrose) delivered via a 0.2 ml Gilmont syringe. On each trial of conditioning, following CS delivery, a 1 M sucrose solution was presented to both antennae to provoke PER, then to the US was presented to the proboscis for consumption. If a subject refused to eat the US, they were force-fed the solution. Subjects responding spontaneously to the conditioned stimulus on the first trial were excluded from analysis. The conditioned response was recorded as a binary variable (PER or no PER) following CS delivery just prior to US presentation.

### 2.5 Statistical analysis

All data analyses were performed using SPSS 21. Pearson’s correlation and a linear regression (lreg) were carried out for the 2-day gustatory mouthparts assay data. The amount consumed gustatory assay was compared using 1-way ANOVA. PER and conditioning data were analysed using logistic regression or a generalized linear model (GLZM) with a Tweedie distribution. *Post hoc* pairwise comparisons for individual treatments were performed using least squares contrasts (lsc).

## 3 Results

### 3.1 100 mM AAs in 1 M sucrose solution affect feeding

**Fig. 1:**
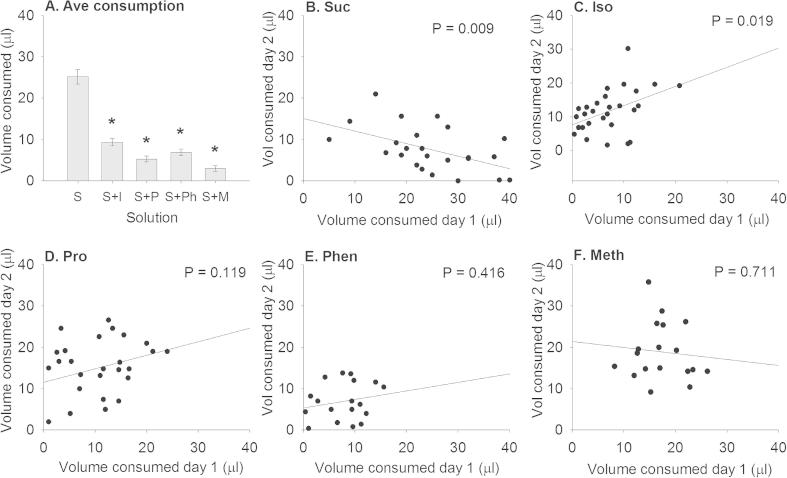
Nutritional state, as determined by pre-feeding bees with specific solutions, influences the amount of food consumed. (A) Bees pre-fed 1 M sucrose solution consumed less of 1 M sucrose solution containing 100 mM AAs. *N*_sucrose_ = 30; *N*_isoleucine_ = 30; *N*_proline_ = 30; *N*_phenylalanine_ = 30; *N*_methionine_ = 30. (B–F) Pre-feeding bees influenced how much food was consumed 24 h later. (B) Bees pre-fed 1 M sucrose ate significantly less sucrose the next day. (C) Bees pre-fed sucrose containing isoleucine ate significantly more isoleucine solution the next day, whereas bees fed solutions containing proline (D), phenylalanine (E), and methionine (F) did not alter their intake 24 h later. *N*_sucrose_ = 25; *N*_isoleucine_ = 28; *N*_proline_ = 27; *N*_phenylalanine_ = 18; *N*_methionine_ = 19.

We first tested how bees reacted when their proboscis (mouthparts) were presented with a sucrose solution containing AAs after PER was elicited (Fig. 1). When applied to the mouthparts, sucrose solutions containing 100 mM AAs inhibited food consumption (Fig. 1A, 1-way ANOVA, solution: *F*_4, 145_ = 73.2, *P < *0.001). Isoleucine reduced the amount consumed by 62% of the response to sucrose; methionine reduced it by 88%.

To test if feeding a sucrose solution containing AAs altered whether bees would feed on sucrose solutions containing AAs 24 h later, bees were fed 1 M sucrose or 1 M sucrose solution containing a 100 mM AA and then their proboscis was presented with the same solution to see how much they would drink. When fed with sucrose, bees were less likely to consume as much sucrose the next day (Fig. 1B, Pearson’s *r* = −0.509, *P = *0.009). However, when fed with a sucrose solution containing isoleucine, they consumed more of the solution 24 h later (Fig. 1C, isoleucine; Pearson’s *r* = 0.441, *P *= 0.019). Bees fed with sucrose solutions containing proline (Fig. 1D), phenylalanine (Fig. 1E), or methionine (Fig. 1F) did not adjust the amount of solution they consumed 24 h later (proline: Pearson’s *r* = 0.307, *P *= 0.119; phenylalanine, Pearson’s *r* = 0.204, *P *= 0.416; methionine: Pearson’s *r* = −0.091, *P = *0.711). To test if AAs stimulated feeding in the absence of sucrose, AA solutions were applied to the proboscis in an ascending concentration series. Sucrose alone was the only solution that demonstrated a consistent increase in amount consumed with concentration (Supplemental Fig. 1, main effect, 1-way ANOVA, solution: *F*_4,145_ = 19.7, *P < *0.001). The 100 mM dose of phenylalanine was slightly phagostimulatory when applied to the mouthparts, but none of the other AAs stimulated consumption at any of the concentrations tested.

### 3.2 Pre-feeding with AAs modulates learning

**Fig. 2:**
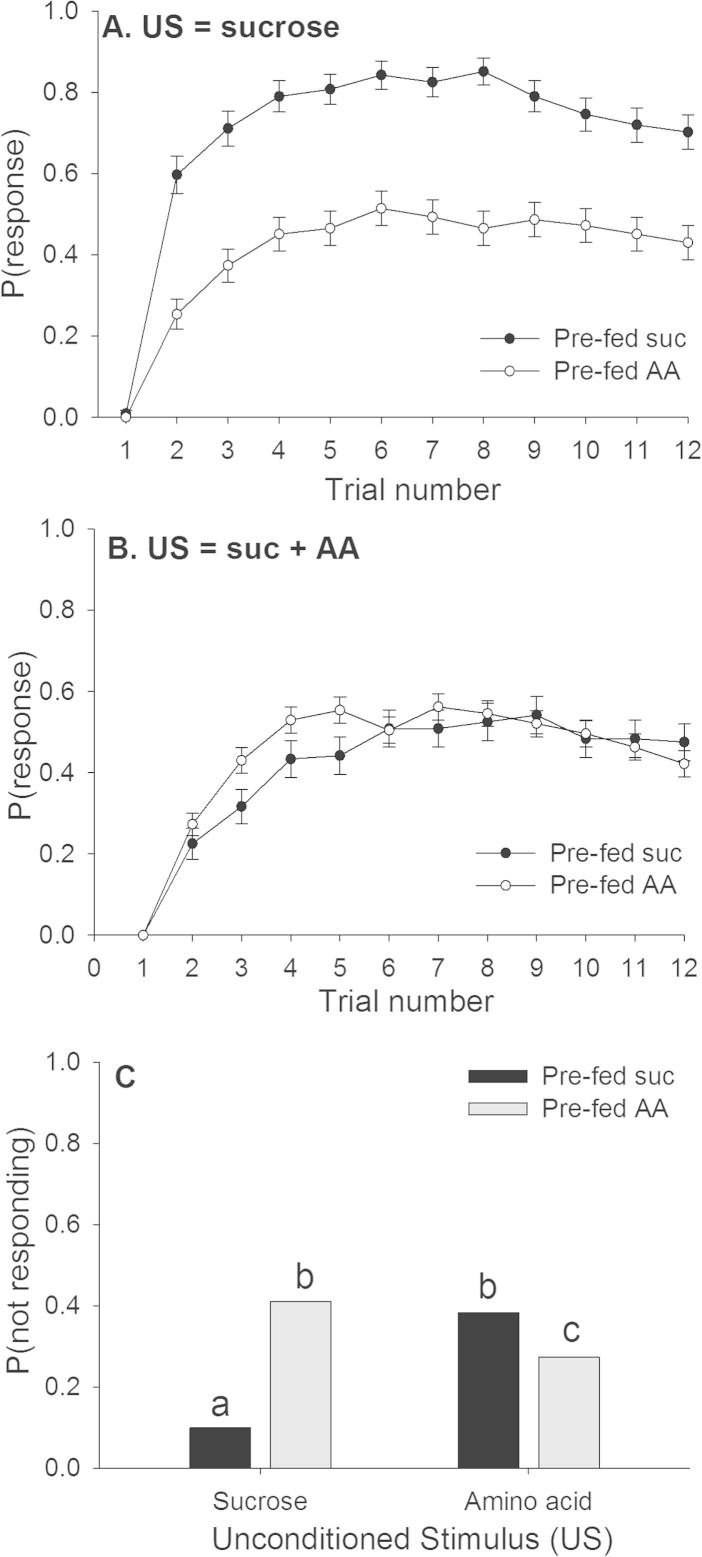
Pre-feeding treatment and US type influenced the number of bees that learned to exhibit conditioned PER during training. (A) Bees trained with a US containing 100 mM AA solutions were less likely to respond during conditioning (AA–S) than those fed with sucrose and then trained with sucrose (S–S). (B) Pre-feeding with sucrose (S–AA) or sucrose containing AA (AA–S) did not influence the response during conditioning to the sucrose-only US. (C) The proportion of bees that did not respond on any trial during conditioning (i.e. proportion non-responsive) depended on both the pre-fed solution and the US. Letters indicate *P* < 0.05 in pairwise comparisons. Note: A–C represents the pooled data from Fig 4. *N*_S–S_ = 91; *N*_S–AA_ = 120; *N*_AA–S_ = 117; *N*_AA–AA_ = 121.

Feeding history influenced the way that bees responded to solutions containing AAs during conditioning, but in a way opposite to our predictions (Table 1). Bees pre-fed sucrose solution 24 h before conditioning quickly learned to express conditioned PER towards an odour signalling a sucrose reward (Fig. 2A). However, those pre-fed sucrose and then trained with a US containing 100 mM AAs had a lower asymptotic level of response, as indicated by the fact that on average, a smaller proportion of the population responded during conditioning (Fig. 2A). In contrast, bees fed a solution containing an AA 24 h prior to conditioning and trained with a solution containing this AA in the US acquired the same asymptotic level of conditioning as those fed sucrose and then conditioned with an AA (Fig. 2B). The responses during conditioning also depended on the AA used in pre-feeding and reinforcement (US) during conditioning (Table 1); this is discussed in Section 3.3 below.

**Table 1:** Repeated-measures GLZM for bees conditioned with rewards containing AAs.

	Type III		
	*χ*^2^	df	*P*-value
Pre-fed trt	14.4	4	<0.001
US	10.8	1	<0.001
Trial number	153	10	<0.001
Amino acid (cov)	4.51	1	0.032
US^∗^Pre-fed	20.6	1	<0.001
US^∗^Trial	8.91	10	0.543
Pre-fed^∗^Trial	12.5	10	0.255
US^∗^Pre-fed^∗^Trial	22.2	10	**0.014**

Analysis conducted on pooled data for all amino acids and pre-fed treatments. Type of amino acid was entered as a covariate.

Relevant model terms are highlighted in bold.

We also examined the proportion of bees in each group that did not respond on any trial during conditioning (‘non-responders’) (Fig. 2C). The percentage of ‘non-responsive’ bees depended on both the pre-feeding treatment and the US (lreg, US^∗^Pre-fed interaction, *χ*_1_^2^ = 5.3, *P *< 0.001) and was not affected by the identity of the AA (lreg, covariate AA, *χ*_1_^2^ = 0.002, *P *= 0.967). In opposition to our original expectations, bees were less likely to respond to a sucrose US if they had been pre-fed a sucrose–AA solution; however, they were less likely to respond to a sucrose–AA solution during conditioning when they had been pre-fed sucrose.

### 3.3 100 mM proline, phenyalanine, and methionine in a sucrose US reduce appetitive learning

**Fig. 3:**
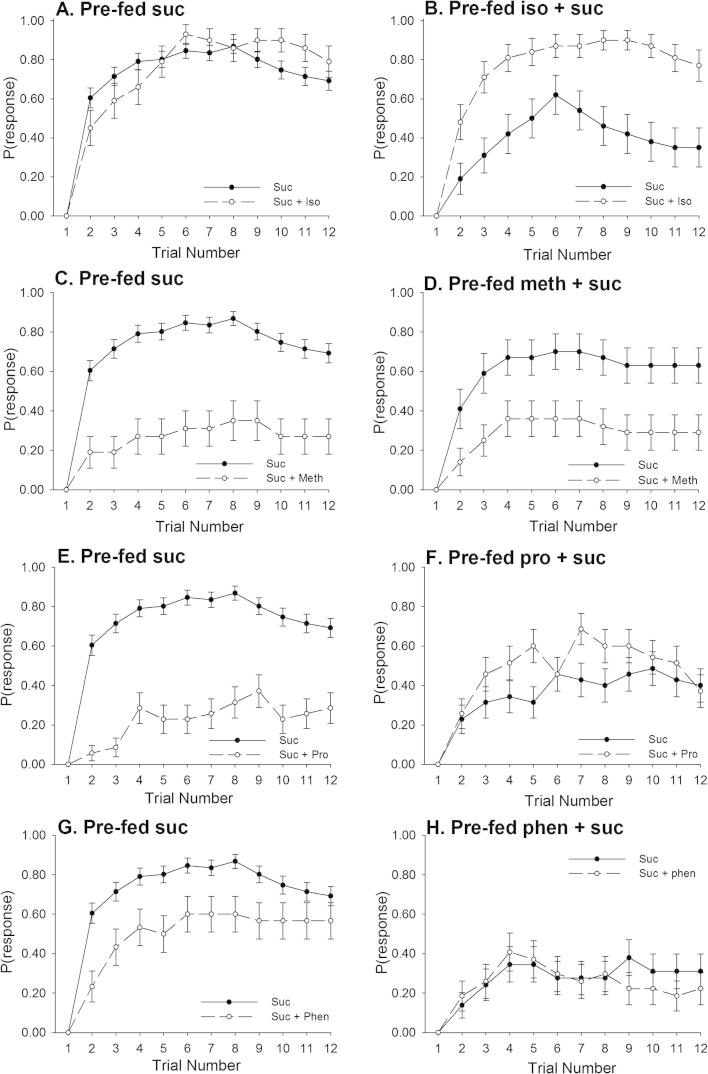
The rate of acquisition during olfactory learning was influenced by both US solution and nutritional state. (A) Bees trained with solutions containing isoleucine learned as well as those trained with sucrose. (B) Bees pre-fed with solutions containing isoleucine (iso) were more likely to respond to this solution than sucrose alone during conditioning. (C and D) Pre-feeding treatment did not have a strong influence on the rate of response during conditioning in bees trained and pre-fed with methionine. (E) Bees trained with solutions containing proline (pro) responded less if pre-fed with sucrose than when pre-fed with sucrose–proline solutions (F). (G) Bees trained with solutions containing phenylalanine (phen) responded less than those trained with a sucrose-only US. (H) Bees pre-fed solutions containing phenylalanine had low rates of response during conditioning to either a sucrose US or a sucrose–phenylalanine US. (Note: the sucrose US curve is the same in figures A, C, E, G, *N*_Sucrose_ = 92). Isoleucine: *N*_S–AA_ = 29; *N*_AA–S_ = 26, *N*_AA–AA_ = 31; Proline: *N*_S–AA_ = 35; *N*_AA–S_ = 35; *N*_AA–AA_ = 35; Phenylalanine: *N*_S–AA_ = 30; *N*_AA–S_ = 29, *N*_AA–AA_ = 27; Methionine: *N*_S–AA_ = 26; *N*_AA–S_ = 27; *N*_AA–AA_ = 28.

Because we found that the responses in Fig. 2A and B depended on the AA used, we did a separate analysis of pre-feeding treatment and US for each AA (Fig. 3). Each AA had a specific effect on learning when it was pre-fed to bees 24 h prior to conditioning. The results of these analyses revealed that isoleucine and methionine influenced learning in distinct ways to proline and phenylalanine (Fig. 3, Table 2).

**Table 2:** Repeated-measures GLZM for bees conditioned with rewards containing single AAs.

	Iso-leucine			Methionine			Proline			Phenylalanine		
	Type III			Type III			Type III			Type III		
	*χ*^2^	df	*P*-value	*χ*^2^	df	*P*-value	*χ*^2^	df	*P*-value	*χ*^2^	df	*P*-value
Pre-fed trt	4.70	1	0.030	0.574	1	0.449	0.411	1	0.521	22.1	1	<0.001
US	8.97	1	0.003	**22.7**	**1**	**<0.001**	11.5	1	0.001	3.35	1	0.067
Trial number	**99.5**	**10**	**<0.001**	50.8	10	<0.001	57.2	10	<0.001	51.3	10	<0.001
US^∗^Pre-fed	**5.31**	**1**	**0.021**	1.046	1	0.306	26.3	1	<0.001	2.05	1	0.152
Pre-fed^∗^Trial	4.75	10	0.907	**18.5**	**10**	**0.048**	17.2	10	0.070	12.1	10	0.280
US^∗^Trial	12.5	10	0.251	9.97	10	0.442	16.9	10	0.075	4.26	10	0.935
US^∗^Pre-fed^∗^Trial	7.37	10	0.692	8.10	10	0.619	**26.8**	**10**	**0.004**	**22.2**	**10**	**0.014**

Relevant model terms are highlighted in bold.

Pre-feeding had a distinct influence on the response to solutions containing isoleucine during conditioning (Table 2, GLZM, US^∗^pre-fed interaction, *χ*_1_^2^ = 5.31, *P *< 0.001). Bees fed sucrose and then trained with a sucrose solution containing 100 mM isoleucine learned at the same rate as those trained with sucrose (Fig. 3A, lsc, *P *= 0.611). Bees fed with 100 mM isoleucine 24 h prior to conditioning quickly learned to associate an odour with sucrose containing isoleucine, but they were less likely to learn an odour associated with sucrose (Fig. 3B, lsc, *P *= 0.006).

Pre-feeding with methionine slightly modulated the conditioned PER, as the average response across all the trials was lower for those pre-fed methionine than bees fed with sucrose (Table 2, Fig. 3C–D, GLZM, Pre-fed^∗^trial interaction, *χ*_1_^2^ = 18.5, *P* = 0.048). However, bees pre-fed and conditioned with solutions containing methionine always had lower average conditioned responses than those trained with a sucrose-only US (Table 2, GLZM, US main effect, *χ*_1_^2^ = 22.7, *P* < 0.001).

In contrast, bees pre-fed and conditioned with solutions containing proline or phenylalanine responded in similar ways (Table 2). When bees were first fed sucrose and then conditioned with a sucrose solution containing 100 mM proline or phenylalanine, they responded at a lower rate on average than bees fed sucrose (Fig. 3E–H, lsc, pro: *P *= 0.035; phenyl: *P *= 0.003). However, if bees had been pre-fed with a sucrose solution containing proline or phenylalanine, their responses to a sucrose-only US during conditioning the next day were relatively low on average and not significantly different to those conditioned with a sucrose–amino acid US (lsc, pro: *P *= 0.246; phenyl: *P *= 0.805).

### 3.4 Pre-feeding with AAs affects appetitive learning

**Fig. 4:**
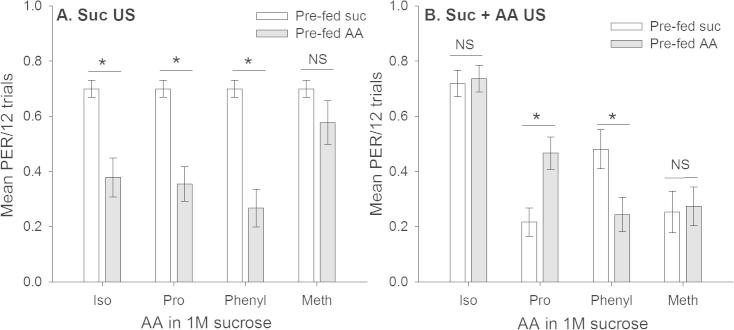
Pre-feeding with solutions containing 100 mM AAs had a complex influence on responses during conditioning. (A) Bees pre-fed a sucrose–AA US were less likely to respond to an odour predicting a 1 M sucrose US. (B) Pre-feeding with a sucrose–AA solution affected responses of bees to a sucrose–AA US in a way that depended on the AA used. Bees trained with isoleucine or methionine containing solutions did not modulate their behaviour to the sucrose–AA US. Bees conditioned with a US containing proline were more likely to respond during conditioning if pre-fed proline, whereas those conditioned with a US containing phenylalanine were less likely to learn if pre-fed a solution containing phenylalanine. Sample sizes as in Fig. 4. ^∗^ Indicates *P* ⩽ 0.05.

We did a separate comparison of the average response over all 12 trials (using the data above) to compare the influence of pre-feeding treatment on the way that the bees responded to US presented during training. This analysis was performed to identify whether there was a significant influence of pre-feeding treatment the asymptotic level of acquisition during conditioning. Bees that had been pre-fed sucrose and then trained with a sucrose US responded on ∼70% of the trials during conditioning (Fig. 4A). These bees responded on more of the trials than bees fed with an AA solution and then trained with sucrose (GLZM, AA main effect, *χ*_4_^2^ = 19.7, *P *= 0.004). Pre-feeding had a more complicated effect on the motivation of bees to learn to associate an odour with a US containing AAs (Fig. 4B): the average response during conditioning depended on both the pre-feeding treatment and the AA used (GLZM, AA^∗^pre-fed, *χ*_3_^2^ = 9.49, *P *= 0.023). Pre-feeding treatment did not alter the response to a US containing isoleucine (lsc, *P *= 0.928) or methionine (lsc, *P *= 0.835). Pre-feeding with proline caused bees to be more likely to learn to associate an odour with a US containing proline than those fed with sucrose (lsc, *P *= 0.021). In contrast, pre-feeding with phenylalanine reduced the likelihood that bees would learn to associate an odour with a US containing phenylalanine compared to a sucrose US (lsc, *P *= 0.051).

## 4 Discussion

All of the AAs we tested were detectable and slightly deterrent when fed to bees at a concentration of 100 mM in 1 M sucrose solution. Bees fed sucrose 24 h prior to conditioning were less likely to learn to perform appetitive PER towards an odour signalling a US containing proline, phenylalanine, or methionine, but bees trained with isoleucine were unaffected. Pre-feeding with an AA–sucrose solution prior to conditioning with a sucrose US uniformly reduced conditioned PER. Bees trained with sucrose containing isoleucine or methionine were unaffected by pre-feeding with AAs, whereas bees fed with proline or phenyalanine were. Bees pre-fed with proline were more likely to learn when reinforced with a US containing proline, whereas those pre-fed phenylalanine were less likely to learn.

The taste of the unconditioned stimulus (US) has a strong influence on the asymptotic level of conditioning during appetitive olfactory PER in honeybees. High concentration sucrose solutions are learned faster (Loo and Bitterman, 1992, Scheiner et al., 1999). Solutions containing a deterrent substance like the toxin, quinine, inhibit PER; as a result, when they are present in a sucrose solution, they reduce appetitive responding towards the US during conditioning and result in an aversive olfactory memory (Wright et al., 2010; Mustard et al., 2012). Amino acids are both phagostimulatory and inhibitory of feeding in insects (Robbins et al., 1965, Shimada, 1978, Lanza, 1988). Whether or not they are inhibitory or excite feeding depends on their concentration (Carter et al., 2006) and on which sensory neurons they excite. For example, in flesh flies and blow flies phenylalanine stimulates sugar-sensing neurons, whereas proline excites salt-sensing neurons in flies (Shiraishi and Kuwabara, 1970, Goldrich, 1973). The 100 mM AA solutions we used were clearly inhibitory of feeding behaviour when bees first experienced them. For this reason, the depression in the conditioned responses of bees pre-fed with sucrose and trained with sucrose–AA solutions is consistent with the interpretation that these solutions are bitter or bad tasting to bees and the bees were learning to avoid odours associated with them. However, we expected to observe this behaviour when the bees were over-sufficient in a specific AA, but we observed it only after bees had been fed with sucrose solution 24 h earlier.

Nutrient balancing requires that animals integrate information about nutritional state with decisions about what foods to consume (Schwartz et al., 2000, Simpson and Raubenheimer, 2012). For this reason, we expected that pre-feeding AA solutions should further reduce PER and appetitive conditioning of PER towards AA solutions 24 h later. However, only bees fed with phenylalanine had reduced responding to phenylalanine the next day. Bees pre-fed methionine did not alter their responses to methionine; they continued to respond as if methionine was aversive in sucrose solution. Unexpectedly, bees pre-fed isoleucine or proline were instead more likely to express conditioned PER when trained with solutions containing these AAs.

These data indicate at least two important characteristics of the feedback about AAs. First, sufficiency of a single AA does not uniformly suppress gustatory/learned responses to this AA and result in aversive learning. This implies that multiple AAs are necessary to signal the protein/AA sufficiency in a way that alters feeding decisions and gustatory sensitivity to AAs (Laeger and Morrison, 2013). Indeed, one of the main differences of our study to previous work on how nutritional state influences sensitivity to AAs in insects was that previous work has always used a basic medium or a mixture of essential and non-essential AAs to alter nutritional state (Simpson and Simpson, 1992, Toshima and Tanimura, 2012). However, we did not test all the AAs that could be incorporated into proteins in insects, and it is possible that others might suppress feeding uniformly.

Surprisingly, bees pre-fed AA solutions and then trained with sucrose were less likely to learn. This is contrary to our original predictions that nutritional sufficiency should drive the phagostimulatory properties of food and hence learning. It is possible that feeding with AAs affects multiple pathways, including those that control hunger state. State of hunger has a strong influence on animal performance in associative learning and memory tasks (Friedrich et al., 2004, Krashes et al., 2009) and is mediated in part by neuropeptide F in insects (Wu et al., 2005, Krashes et al., 2009). In general, hunger is produced in animals by depriving them of carbohydrates. We estimate that the AA solutions provided 10% more calories than sucrose alone (May and Hill, 1990), so it is possible that this population was less hungry than those fed sucrose alone. However, our data may also indicate that the same neuronal mechanisms that regulate and respond to carbohydrates also respond to AAs. These mechanisms may not be the same in insects and mammals. For example, a recent study in rats showed that solutions containing arginine, lysine, or glutamic acid administered intragastrically on their own and not in the presence of other AAs reliably suppressed feeding on rat chow (Jordi et al., 2013). Unlike the AAs in our study, the other ‘proteogenic’ AAs did not suppress rat feeding.

Second, our data for bees pre-fed with AAs also shows that some AAs are post-ingestively reinforcing to bees. This was especially apparent for bees fed isoleucine, but was also true for those fed proline. When they had been fed with sucrose containing isoleucine, these bees ate more isoleucine laced solutions the next day and also showed improved learning performance 24 h later towards solutions containing isoleucine. Indeed, these bees’ responses exceeded those of bees trained only with sucrose. Even more surprising is the fact that the bees learned to avoid odours paired with solutions containing proline if they had consumed only sucrose day the before, but were more likely to learn to associate an odour with proline in sucrose solution if they had been fed proline the day before. This suggests that the post-ingestive reinforcement effects of proline were greater than the pre-ingestive ‘bad taste’ caused by the AA and allowed the bees to overcome their initial aversion after a period of time following proline consumption.

Several recent studies have shown that feeding bees with sucrose during conditioning (Wright et al., 2007) or training Drosophila with solutions containing metabolisable sugars (Burke and Waddell, 2011, Fujita and Tanimura, 2011, Miyamoto et al., 2012) improves olfactory memory formation and food choice. These experiments, coupled with others showing that toxins cause conditioned aversions towards odours (Wright et al., 2010) indicate that the brain senses nutrients after they have been eaten. How this occurs in animals has not been established, although a recent study in flies has shown that knock-out of a glucose transporter and sugar-sensing gustatory receptors alters food preference in adult Drosophila (Dus et al., 2013). As in mammals (Karnani et al., 2011, Jordi et al., 2013), our data strongly indicates that neurons in pathways involved in the reinforcement of visual or olfactory stimuli with gustatory input in the brain are also responsive to specific AAs. Further research is necessary to identify which AAs are reinforcing, how AAs influence olfactory memory formation, and whether or not the concentration of the AA affects its value as a post-ingestive reinforcer during learning.

Bees encounter free AAs in nectar when they are foraging. For this reason, it is reasonable to expect that free AAs in a solution of sugars could influence the bee’s olfactory learning and memory. The four AAs that we used to do these experiments are far from exhaustive, and are only a small subset of the AAs encountered in nectar. In fact, it is common for nectar to have 10–20 different AAs present, albeit in the micro-nanomolar range of concentrations (Petanidou et al., 2006). We chose to test phenylalanine and proline because they are commonly present at high concentrations in floral nectar (Petanidou et al., 2006). Proline is used as fuel by bee flight muscles (Micheu et al., 2000) and free-foraging honeybees prefer to collect sucrose solutions containing ∼4–6 mM proline in 1 M sucrose (Carter et al., 2006). The AAs, phenylalanine, isoleucine and methionine, are essential AAs that are important for protein synthesis in bees (de Groot, 1952); their intake is likely to be strictly regulated by bees for this reason (Paoli et al., 2014). What is surprising from our study is that these three essential amino acids all affected learning in different ways. We predict that future work that identifies the link between the mechanisms for post-ingestive sensing of amino acids in the brain will show that only some essential amino acids are necessary to signal ‘protein’ sufficiency – and that one of these will be isoleucine.
